# Atrioventricular Nodal Dysfunction Secondary to Chronic Alcohol Abuse

**DOI:** 10.7759/cureus.35595

**Published:** 2023-02-28

**Authors:** Bradley Casey, Taylor Harper, Amol Bahekar, Divyang Patel, Raviteja Guddeti

**Affiliations:** 1 Internal Medicine, Cape Fear Valley Medical Center, Fayetteville, USA; 2 Cardiology, Cape Fear Valley Medical Center, Fayetteville, USA; 3 Cardiovascular Medicine, Creighton University School of Medicine, Omaha, USA

**Keywords:** intubated, pacemaker, alcohol abuse, supraventricular bigeminy, complete heart block

## Abstract

Atrioventricular (AV) nodal conduction disorders occur when the AV conduction pathway is refractory due to functional or organic reasons, resulting in a delay or complete blockage of atrial impulses to the ventricles. One of the causes of nodal dysfunction includes chronic alcohol abuse and excessive binge drinking. We are presenting a case where a chronic alcoholic was binge drinking due to a loss of a close friend, which resulted in nodal dysfunction and multiple cardiac rhythms, including supraventricular bigeminy, sinus bradycardia, significant sinus pauses, and complete heart block. He eventually got a single-chamber permanent pacemaker and endorsed that he quit drinking alcohol when he was discharged. He followed up with cardiology after discharge, and his pacemaker interrogation showed that he has been without any type of cardiac arrhythmias.

## Introduction

Atrioventricular (AV) nodes, located at the Koch triangle in the heart, can become dysfunctional, causing electrical conduction to be hindered [1]. When consumed excessively, alcohol consumption is associated with heart rhythm disorders, which have been collectively known as "holiday heart syndrome" [2]. In binge drinkers, AV conduction disturbances are rarer but clinically significant [2]. Supraventricular bigeminy can be seen as a cause of AV nodal dysfunction, and the phrase escape-capture bigeminy has been used in place of pseudo-reciprocal rhythm [3]. Here, we present a rare case of a patient that is a chronic alcoholic and has been having multiple syncopal episodes at home. The family thought the patient was passing out at home due to his excessive alcohol consumption, but we will later find out it was likely secondary to his underlying arrhythmias as opposed to his alcohol abuse.

## Case presentation

A 73-year-old male patient with a past medical history of alcoholic cirrhosis presented to the emergency department after the family discovered him at home sitting in his own feces and urine. The family reported that he was confused and not responding to any of their questions. The family reported that over the past several weeks, he had been falling down while ambulating, but they were relating it to his copious amounts of alcohol consumption. The family did say that during one of his syncopal episodes, he was unconscious for approximately seven minutes, and they denied any urine or bowel incontinence. The family called emergency medical services (EMS) and when they arrived, they found him to be hypoxic with oxygen saturation in the 80s while on room air. His oxygen saturation improved to the low 90s on a 3-liter nasal cannula. EMS reported that his heart rate was sinus rhythm in the '80s (no rhythm strip available). Upon arrival at the emergency department, he was hypothermic with a temperature of 87 degrees Fahrenheit and bradycardic in the low 40s (no rhythm strip available). He was given 0.5 mg of atropine and 500 cc bolus of lactated ringers. He was hypotensive with a blood pressure of 72/38 mmHg by manual blood pressure cuff after the 500 cc fluid bolus, and he was started on Levophed with the broad-spectrum antibiotics vancomycin and cefepime. He was still altered and not responding to verbal or painful stimuli, so he was intubated. When the family arrived at the emergency department, they reported that he normally would drink one pint of vodka daily, but they believed he had been binge drinking lately due to the passing of a close friend. They were unsure when his last drink was, and EMS did not report any alcoholic beverages around the patient's chair when they got into the house. The patient's initial blood work can be seen in Table 1.

**Table 1:** Initial laboratory values of the patient

Laboratory Test	Reference Range	Patient's Lab
Complete Blood Count		
White Blood Cell Count	4.5 - 12.5 x10*3/uL	8.6 x10*3/uL
Hemoglobin	12.0 - 16.0 g/dL	7.9 g/dL
Mean Corpuscular Volume	81.0 - 99.0 fL	100.8 fL
Platelets	150 - 450 x10*3/uL	70 x10*3/uL
Comprehensive Metabolic Panel		
Sodium	136 - 145 mmol/L	135 mmol/L
Potassium	3.5 - 5.1 mmol/L	3.9 mmol/L
Bicarbonate	21 -32 mmol/L	14 mmol/L
Chloride	98 - 107 mmol/L	104 mmol/L
Blood Urea Nitrogen	7 - 25mg/dL	18 mg/dL
Creatinine	0.60 mg/dL	1.07 mg/dL
Glucose	74 - 106 mg/dL	111 mg/dL
aspartate aminotransferase	15 - 37 U/L	166 U/L
alanine transaminase	12 - 78 U/L	72 U/L
glomerular filtration rate	>60.0 mL/min/1.73m*2	>60.0 mL/min/1.73m*2
Albumin	3.5 - 5.7 g/dL	2.3 g/dL
Magnesium	1.9 - 2.7 mg/dL	1.3 mg/dL
Arterial Blood Gas		
Arterial pH	7.35 - 7.45 pH	7.19 pH
Oxygen	75 - 100 mmHg	85 mmHg
Carbon Dioxide	35.0 - 45.0 mm Hg	49 mm Hg
Bicarbonate on Arterial Blood Gas	22.0 - 26.0 mEq/L	13.8 mEq/L
Other Blood Tests		
Ammonia	11 - 32 umol/L	43 umol/L
Thyroid Stimulating Hormone	0.358 - 3.740 uIU/mL	2.412 uIU/mL
Free T4	0.76 - 1.46 ng/dL	1.07 ng/dL
Ethanol Level	<3 mg/dL	<3 mg/dL
High Sensitivity Troponin	2 -20 pg/mL	23 pg/mL
3 Hour High Sensitivity Troponin	2 -20 pg/mL	19 pg/mL
B-Type Natriuretic Peptide	< 100 pg/mL	468 pg/mL
Lactic Acid	0.5 - 2.0 mmol/L	1.3 mmol/L

**Figure 1:**
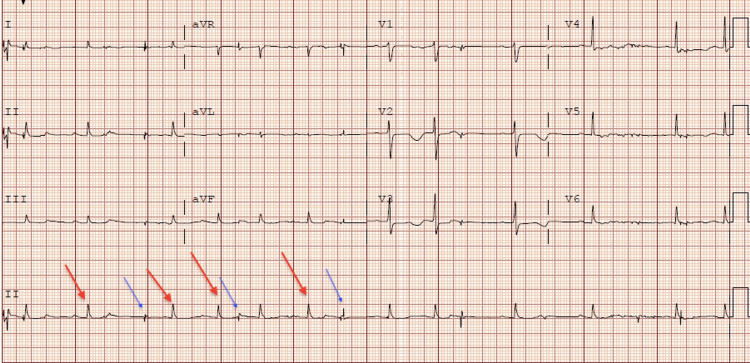
Initial electrocardiogram showed sinus rhythm 72 beats per minute, supraventricular bigeminy, and low voltage The red arrows in the electrocardiogram show the patient's normal conducted QRS followed by the blue arrows, which depict the supra-ventricular ectopic beats.

**Figure 2:**
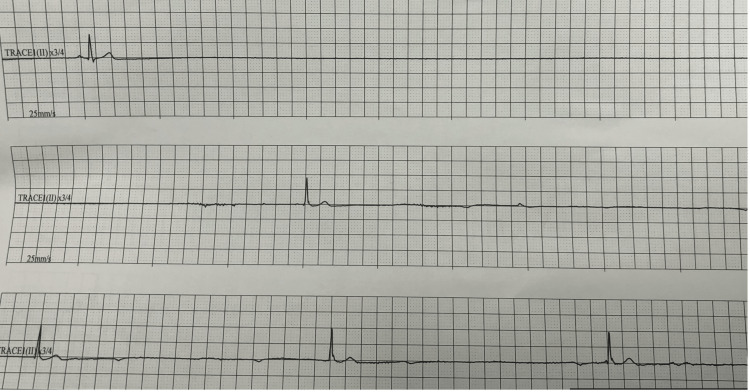
Rhythm strip captured from the intensive care unit showing a sinus pause in rows 1, 2, and 3

**Figure 3:**
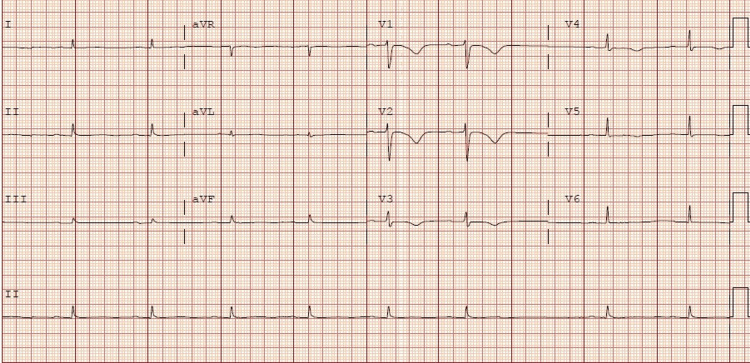
Repeat EKG after telemetry called to report a complete heart block that showed sinus bradycardia of 55 beats per minute, sinus pause of nearly 2 seconds, prolonged PR interval, and low voltage in inferior leads

Due to the patient's initial presentation, he had a CT scan of the head without contrast and the results were unremarkable. Initial electrocardiogram (EKG) showed a sinus rhythm rate of 72 beats per minute, supraventricular bigeminy, and low voltage (Figure 1). While in the intensive care unit, it was noted on telemetry that he was experiencing sinus pauses, with the longest being seven seconds (Figure 2). The patient then proceeded to have a Mobitz type I AV block, as well as frequent blocked premature atrial contractions (telemetry strip not available). As the patient was getting prepped for a temporary transvenous pacemaker (TTP), telemetry called and reported that the patient was in complete heart block for approximately 16 seconds (no rhythm strip available). Repeat EKG (Figure 3) showed a sinus bradycardia rate of 55 beats per minute, sinus pause of nearly 2 seconds, prolonged PR interval, and low voltage. TTP was placed, and the electrophysiology team was requested to evaluate the patient. All the patient's electrolyte abnormalities were corrected and no infectious process was present so antibiotics were discontinued. There was no history of coronary artery disease, and high-sensitivity troponin was the upper limit of normal. The patient underwent a cardiac transthoracic echocardiogram, which showed preserved ejection fraction greater than 55%, normal right ventricular function, no diastolic dysfunction, and no wall motion abnormalities. The patient's heart rate remained sinus with intermittent atrial bigeminy on telemetry, and he only had two episodes that telemetry picked up as paced rhythm. The patient eventually had a single-chamber pacemaker placed, and he was extubated and transferred out of the intensive care unit. The patient was followed up in the outpatient setting and reported that he stopped drinking alcohol entirely after the hospitalization. Prior to the hospitalization, he could not recall the exact amount of alcohol he was drinking but reported it was over a pint per day. At his follow-up appointment, his pacemaker was interrogated and revealed no further cardiac arrhythmias.

## Discussion

Currently, there are three mechanisms for which escape-capture bigeminy can occur: (1) A long intersinus interval at the lower pacemaker (ventricular) level as a result of an S-A block, (2) an escape-capture bigeminy due to an AV block, (3) an escape-capture bigeminy due to reversed reciprocal rhythm [3]. AV block following acute alcohol intoxication has been studied by Arthur H van Stigt and colleagues. They determined that second and third-degree AV blocks were the most commonly seen, and once the patients became sober again, they returned to sinus rhythm [2].

Electrocardiogram findings alone, particularly sinus bradycardia, do not indicate the presence of nodal dysfunction; node dysfunction is defined by the coexistence of symptoms and electrocardiogram results [4]. Current electrocardiogram findings to look for in nodal dysfunction include: (1) severe sinus bradycardia, (2) pauses, arrests, and blocks that can occur with or without escape rhythms, and (3) tachy-brady syndrome [4].

A patient with nodal dysfunction must first be evaluated for hemodynamic stability [4]. Clinical practitioners should follow the advanced cardiac life support (ACLS) protocol for symptomatic bradycardia in unstable patients, and atropine should be tried first with 0.5 mg repeated every three to five minutes for a total dose of 3 mg. Transcutaneous pacing and chronotropic agents should not be delayed for atropine treatment [4]. In stable patients, clinicians should look for reversible causes to determine if there is a reversible underlying cause. Currently, there are no guidelines regarding asymptomatic patients, but they should be observed first before proceeding to a permanent pacemaker [4]. Patients that are symptomatic with sinus node dysfunction will require a permanent pacemaker, either a single-chamber atrial pacemaker or a dual-chamber pacemaker [4].

Chronic heavy drinking has been linked to the development of heart arrhythmias in certain individuals [5]. Alcohol's arrhythmogenic effects are still not clearly defined sufficiently in literature, however, several mechanisms have been proposed [5]. Delays in conduction may be caused by subclinical cardiac muscle damage brought on by long-term excessive alcohol abuse [5]. Additionally, aberrant electrolytes, poor vagal heart rate control, repolarization abnormalities with longer QT intervals, or sleep apnea may be factors, as well as the hyperadrenergic condition of drinking and withdrawal [5]. The American College of Cardiology did a review of nearly 900,000 cases for 12 years and revealed a daily alcoholic drink consumption risk of an abnormal heartbeat increase of 8% [6]. One of the most common arrhythmias seen with binge drinking as well as chronic alcohol consumption is atrial fibrillation [7]. It is likely that our patient's cardiac arrhythmias were all related to his chronic alcohol abuse, especially with his recent binge-drinking episode.

## Conclusions

Here, we presented an interesting case of a patient that developed a high-degree AV block secondary to his alcohol use. While hospitalized, he experienced several different heart rhythms, including sinus bradycardia, long sinus pauses, complete heart block, and supraventricular bigeminy. He was treated with a single-chamber permanent pacemaker, and once he quit consuming alcohol, there were no more reported arrhythmias on his follow-up pacemaker interrogation. When he initially presented, his family thought his presentation was from excessive alcohol consumption, and it would be very easy to become closed-minded on this diagnosis. Our job as clinicians is to explore all possibilities for the cause of a patient's presentation. This case also enforces the importance of discussing alcohol cessation with patients as their arrhythmias could be resolved with alcohol cessation.
